# Surface Modification of PET Fabric by Graft Copolymerization with Acrylic Acid and Its Antibacterial Properties

**DOI:** 10.5402/2011/265415

**Published:** 2011-04-07

**Authors:** M. Abdolahifard, S. Hajir Bahrami, R. M. A. Malek

**Affiliations:** Textile Engineering Department, Amirkabir University of Technology, 424 Harez Avenue, Tehran 15914, Iran

## Abstract

Graft copolymerization of acrylic acid (AA) onto Poly(ethylene terephthalate) (PET) fabrics with the aid of benzoyl peroxide was carried out. The effect of polymerization parameters on the graft yield was studied. Percent grafting was enhanced significantly by increasing benzoyl peroxide (BP) concentrations up to 3.84 g/lit and then decreased upon further increase in initiator concentration. Preswelling of PET leads to changes in its sorption-diffusion properties and favors an increase in the degree of grafting. The antibiotics treated grafted fabrics showed antibacterial properties towards gram-positive and gram-negative microorganisms. FTIR and SEM were used to characterize AA-grafted polyester fabrics.

## 1. Introduction

Poly(ethylene terephthalate) (PET) is commercially one of the most important and successful engineering polymers and is widely used in the form of fiber, films, and molded articles. They are highly crystalline, markedly hydrophobic and do not contain chemically reactive groups, which makes it resistant to straight chemical modification. However, chemical modification by graft copolymerization is an important method to improve its dyeability, antistatic properties, moisture regain, or impart antibacterial properties to the fibers. A number of patents or studies have been published concerning the grafting of various vinyl monomers such as styrene, methmethacrylate, methacrylic acid, acrylonitrile, and acryl amide [1–3]. 

Sacak and Oflaz studied the graft copolymerization of acrylic acid on PET fibers using the free radical initiator [4]. The effect of various copolymerization parameters on the graft yield has been investigated. An increase in the reaction temperature increases the graft yield. The activation energy of the grafting was calculated to be 9.9 kcal/mol. Many researchers used radiation for initiating the reaction of grafting [5–10]. The use of this method to initiate the graft copolymerization on PET fibers is imposed by the fact that these fibers show a high resistance to chemical activators.

Recently, some papers have dealt with chemical and thermal initiation of vinyl monomers grafting on polyester fibers. Most of the experiments were focused on changes in physical and mechanical properties of grafted PET fibers. Acrylic acid was grafted onto polyester fabrics by the electron beam preirradiation method [11]. The acrylic acid-grafted polyester fabrics were found to have good water vapor permeability and excellent water impermeability. Changes of the diameter and the surface structure of fabric fibers shown in SEM photos make it clear that a layer of grafted poly(acrylic acid) was formed on the surface of these PET fibers [12]. Methacrylic acid was grafted on poly(ethylene terephthalate) fibers using benzoyl peroxide. It is reported that the graft yield increased when the reaction temperature increased up to 85°C and decreased with further increase in the temperature. The increase in the concentration of monomers was found to increase the graft yield [13]. 

Thermosensitive membranes by radiation grafting of acrylic acid/N-isopropyl acrylamide binary mixture on PET fabric was reported By Gupta et al.; a binary mixture of N-isopropyl acrylamide (NIPAAm) and acrylic acid (AA) was grafted on polyester fabric as a base material to introduce thermosensitive poly(N-isopropyl acrylamide) pendant chains having LCST lightly higher than 37°C in the membrane. The influence of ferrous sulfate, radiation dose and monomer composition on the degree of grafting was investigated [14]. 

Antibacterial poly(ethylene terephthalate) was reported to be obtained by grafting the yarn with acrylic acid and then treating it with cephalosporin-type antibiotic. Two-stage effective methods for obtaining poly(ethylene terephthalate) yarn with antibacterial properties were reported. In this method, the carboxylic group was incorporated into fibers by grafting poly(acrylic acid) polymerization followed by impregnation of the fibers with an antibiotic which belongs to the cephalosporins with Biotrakson as trade name [15]. Stephen Michielsen and his coworker studied the surface modification of fibers via graft-site amplifying polymers. They said that the suitability of fibers for a particular enduse depends on several factors including strength, modulus, extensibility, chemical resistance, size, shape, and surface tension. All these properties, except surface tension and shape, depend on the properties of the polymer that make up the fiber. Considerable effort has been made to optimize the polymer morphology and its chemical makeup to obtain the desired properties. However, for many applications, the critical property that determines the fiber's suitability depends primarily on the surface, such as surface tension, friction, adhesion, and antimicrobial activity. Traditionally, the choice of a fiber for a particular application has been to choose the fiber based on its bulk properties and price. If the surface properties are not adequate, a topical treatment would be applied. However, after cleaning the fibrous material, much of this topical treatment is lost [15].

Bucheñska has investigated the graft copolymerization of acrylic acid on polyester fibers using benzoyl peroxide. The influence of the main parameters of grafting, the effect of additives on the degree of grafting, and the amount of homopolymer formed during the process have been determined. Furthermore, the values of apparent activation energy have been calculated. Also, the influence of the degree of grafting on the moisture sorption and swelling of modified fibers has been determined. By an additional treatment of the grafted fibers with antibiotics, it is possible to provide the fibers with antibacterial properties. Liberation of antibiotics from fibers into solutions has been examined and mathematically described. Several attempts to immobilize antibiotics in PET fibers by impregnating them with biocide solutions have not, so far, led to their practical utilization for such substances are easily and quickly washed out from the treated fibers. Furthermore, attempts to combine antibacterial agents with PET fibers through chemical bonding have also failed due to the absence of suitable functional groups that could form a chemical bond with a biocide compound. This explains the need for introducing into PET fibers proper functional groups, which in turn would be able to combine an appropriate antibacterial agent. The methods consist of preliminary sulphonation followed by treating the fibers with an appropriate antibiotic. A major defect of this method, along with rather a complicated procedure of sulphonation, is, first of all, the decrease in the strength of fibers. These disadvantages can be eliminated by employing another method to modify PET fibers. Such a process as the one used in the study consists of introducing into fibers, instead of sulphonic, carboxylic groups by PAA or poly(metacrylic acid) grafting which, in turn, can be attached to alkaline antibacterial substances by a chemical bond. In this case, grafting has been achieved in a heterogeneous system in the presence of benzoyl peroxide as an activator. Afterwards, antibiotics such as crystalline penicillin, neomycin, and gentamycin will be combined with the grafted PET fibers [16].

Grafting onto polyester fibers has been reported by Rao. The kinetics of grafting of acrylonitrile, acrylic acid, and vinyl acetate on to polyester fiber by catalytic initiation and radiation were studied. The energy of activation determined for acrylic acid grafting by the catalytic method was 10.7 kcal/mol and for vinyl acetate grafting by the radiation method, 11.7 kcal/mol. In the case of acrylonitrile grafting by the catalytic method, the rate of grafting decreased with increase in grafting temperature, showing the differential behavior of the precipitating type of polymer from that of homogeneous polymerization [17].

Hebeish et al. studied the graft copolymerization of acrylic acid on polyester fibers. They used H_2_O_2_ as initiator and benzoyl alcohol as reaction medium. They researched the effect of initiator and monomer concentration, reaction time, and temperature as well as addition of metallic salt to the polymerization, and they also researched that percent grafting was significantly enhanced by increasing H_2_O_2_ concentration up to 100 mequiev/L and then decreased upon further increase in initiator concentration [18]. Recently, glycidyl methacrylate (GMA) monomer was used for photo-induced surface grafting of PET fabric. This was followed by a padcuring treatment using 1-hydroxy ethylidene-1,1- diphosphonic acid (HEDP), and sulfamic acid (H_2_NSO_3_H) [19]. This grafting and treatment significantly improve the flame retardancy and greatly reduce the dripping tendency of PET fabric. Ping et al. reported grafting of n-butyl acrylate and styrene comonomer on Poly(ethylene terephthalate) (PET) film through gamma-ray- induced graft copolymerization [20]. 

In this paper, poly(ethylene terephthalate) fabric was grafted with acrylic acid using benzoyl peroxide as initiator. The grafted fabrics were characterized. The effect of various grafting parameters such as temperature, time, and concentration of monomer as well as initiator on the grafting yield has been investigated. The grafted fabric was then treated with antimicrobial such as lrgacid LP-10 & DP-300. The antimicrobial and dyeing property of the treated fabric was investigated.

## 2. Materials and Methods

### 2.1. Materials

Poly(ethylene terephthalate) woven fabric of serge pattern with warp and weft (37, 26) in one square centimeter was used. Fabrics were washed with a nonionic detergent Ultravon GP which was obtained from Ciba Company and sodium carbonate. Acrylic acid with the boiling point of 139°C was obtained from Merck Co. It was freed from the inhibitor (hydroquinone) by distillation under low pressure using pure nitrogen. Benzene and benzoyl peroxide which were crystallized from methanol-chloroform mixture and dried under vacuum over P_2_O_5_ were obtained from Merck Co. Other chemicals such as Irgasol NA, Univadin PB, Maxillon Red GRL (from Ciba Co.) and penicillin (Pe), neomycin (Ne), gentamycin (Ge) as gentamycin, Bacteria *Escherichia coli* (*E. coli*), *Staphylococcus aureus*, and Micrococcus luteus, Mohr salt and ZnCl_2_ were laboratory grade.

### 2.2. Grafting Procedure

Grafting was carried out using eight different procedures. *In the first procedure*, active centers on the PET yarn were formed by treatment with 5% (wt/v) BP solution in toluene for 30 min at 50°C. Next, the excess of the BP solution was squeezed out and traces of toluene were removed by evaporation for 15 min at 85°C. The pretreated yarn sample was placed into a 1000 ml reactor. First, water and dispersing agent (Irgasol Na) were added to the reactor, and temperatures were raised to 80°C. AA was then added to the reactor. The grafting time was 60 min after which the reaction was stopped and the samples were removed and washed with boiling water for 4-5 hr to remove the traces of monomers and oligomers. But the grafting percentage was not high. *Method no. 2:* In this method, before grafting, the samples were impregnated with Univadin PB for swelling. The samples were boiled for 1 hr in swelling agent solution with L : R 90 : 1, and after that we followed method no. 1 for grafting. But with this method, also graft percentage was too low. *No. 3:* Samples were treated with NaOH (10%) with L : R 120 : 1 for 2 hr in boiling temperature, and their weight decreased. Method no. 1 was used for grafting. But grafting percentage was low in this procedure as well. *No. 4:* At first, samples were impregnated in AA, water and dispersing agent in reactor and temperature raised to 70°C, and benzoyl peroxide solution in benzene (5% wt/v) was added to the reactor. Grafting time was 1 hr. In this method, the grafting percentage was low. *No. 5:* Samples were swelled in AA solution with water (22%) for 16 hr and 24 hr at 50°C, and after that benzoyl peroxide solution in benzene (5% wt/v) was added to the reactor. The grafting time was 1 hr, but the grafting yield was low.

*No. 6:* Samples were treated with dichloroethane at 20°C for 60 min, and then benzoyl peroxide solution in Benzene (5% wt/v) was added to the reactor. Grafting was carried out for one hour. The grafting percentage was low in this method as well. *No. 7*: Samples were boiled in Univadin PB (4 g/lit, 2 g/lit) for 1 hour, and after that they were washed with water, and then benzoyl peroxide solution in Benzene (5% wt/v) was added to the reactor. The grafting time was 1 hr, but in this method the grafting percentage was low. *No. 8:* The PET fabrics were kept in boiling water containing Univadin. This will let the fabric swell. Then, AA which had been mixed with water (10%) was added to the reactor. The fabric was kept for another 10 minutes to swell under boiling. 0.1 g CuCl_2_ which was dissolved in water was added to the reactor, and immediately Irgasol NA was added too. Benzoyl peroxide was then added to the reactor (0.33 g in l0 CC of Benzene). After 2.5 hr, the reaction was stopped, and the fabrics were removed and washed 8 times with boiling water to remove the traces of monomer and oligomers. The washed fabric was then dried to a constant weight. The grafting percentage was higher than other procedures; therefore, this method was selected. The graft percentage was calculated from the following equation:

(1) $$\frac{W-W_{o}}{W_{o}}\times 100,$$

whereas *W* = weight of the rubric after grafting, *W_o_* = weight of the fabric before grafting.

### 2.3. Antibacterial

**Scheme 1:**
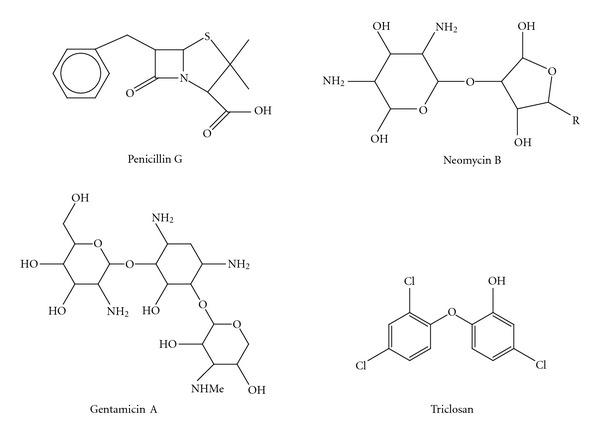


Three antibiotics, namely, penicillin, Neomycin, and gentamycin, were attached to the grafted fabrics, and the cell cultures were carried out on the fabrics. The growth of *Escherichia coli*, *Staphylococcus aureus* and *Micrococcus luteus* on the fabric was investigated. Antimicrobial such as lrgacid LP-10 was also used to study the antimicrobial properties of the grafted fabric. Fabrics with various degrees of grafting were impregnated in a solution of antibiotics Pe, Ne, Ge at 65°C for 1 hour under various conditions of changing the concentration, time, and temperature. Grafted samples and untreated polyester fabrics were treated with antimicrobial solutions (Irgacid Lp 10) and were washed 5 times and dried. Samples were kept in culture media and microbes, that is, *E. coli*, *S. aureus* and *M. luteus*. The fringes were measured after 24 hr at 40°C (see Scheme 1).

### 2.4. Dyeing

Polyester fibers are highly crystalline, markedly hydrophobic, and not containing chemically reactive groups. Hence, this material is not easily penetrated by dyes of large molecular dimensions and cannot combine with dye anions or actions. Certain desirable properties (e.g., dye ability with basic, direct, and other classes of dyes; improvement in antistatic properties; increase in moisture regain) can be imparted in polyester by grafting it with monomers just as AA. We have used 0.5% (owf) Maxillon Red GRL (CI. Basic Red 46) to study the dyeing behavior of the PET fabrics.

### 2.5. Characterization

The FTIR spectrum of AA-grafted PET fabrics was recorded using a Nicolet Nexus model 640 spectrophotometer, with KBr disc. The samples of grafted PET fabrics cut with a microtome were prepared in the form of tables. Thermal properties of the samples were investigated using a DSC (IA instrument). The samples were kept in aluminum pan after accurate weighing. The heating was applied from room temperature up to 300°C with a heating rate of 10°C/min in air atmosphere. The surface characteristics of grafted and ungrafted filaments of fabric were studied with the help of SEM (XL30-SFEG, FEI/Philips) to analyze changes in surface morphology. The filaments taken out of the fabric were first coated with a gold layer to provide surface conduction before scanning.

## 3. Results and Discussion

**Figure 1:**
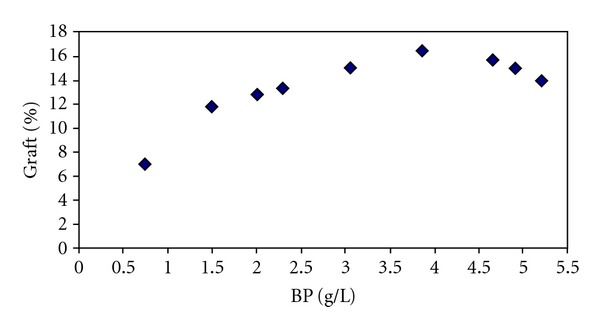
The effect of initiator concentration on the grafting yield.

To study the effect of initiator concentration, the amount of benzoyl peroxide (BP) was changed from 0.76 to 471 g/lit, and its effect on the grafting yield was investigated. Figure 1 shows the change in initiator concentration and its effect on grafting yield. With increasing the BP concentration up to 3.84 g/lit, there is a sharp increase in the grafting %. But after that, the slope of the graft reduces. With increasing the BP concentration, the active sites on the fabric increase which results in an increase in the grafting %. However, after 3.84 g/lit of initiator, the extra free radicals remain in the bath and therefore the monomers get polymerized in the bath which result in increase in the homopolymerization concentration. This was examined and understood after washing the fabric for several hours removing the homopolymers from the fabric.

**Figure 2:**
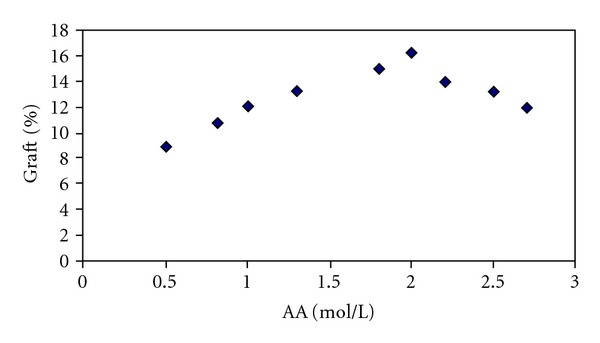
The effect of Acrylic acid monomer concentration on the graft yield.

The concentration of monomer was varied from 0.5 to 9.5 moles in the bath, and its effect on the grafting yield was investigated.  Figure 2 shows the effect of AA concentration on the grafting yield.

As the monomer concentration increases, the number of monomeric radicals which seat on the polyester substrate due to chain transfer increases. But after certain values of monomer concentration due to physical absorption of monomer on the fiber and formation of homopolymer on the fiber surface, the grafting yield is reduced.

**Figure 3:**
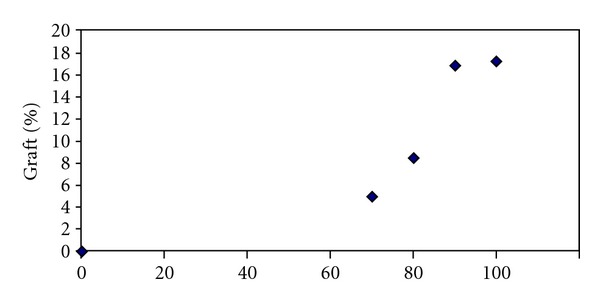
The effect of temperature on grafting of AA on polyester fabric on the grafting yield.

Increasing the temperature, however, results in increasing the rate of decomposition of the initiator. Therefore, with increasing the temperature, the rate of formation of free radicals on the main polymer increases. Increasing the temperature, however, increases the molecular mobility, initiator free radical, and active chain of PAA which causes better swelling or the speed of prorogation on the fiber and will therefore increase the graft yield (Figure 3).

**Figure 4:**
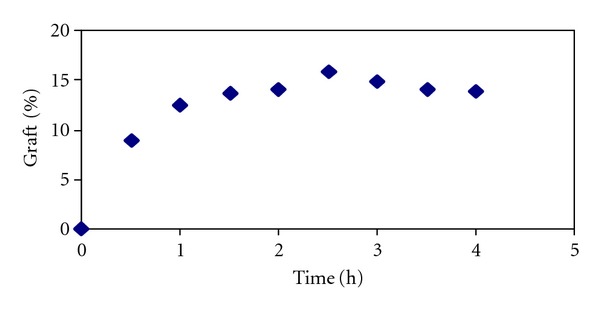
Effect of time of reaction on the grafting yield.

The effect of time on the graft yield is presented in Figure 4. Grafting was carried out for 1–4 hrs. With increasing the time of reaction, the graft yield increased. The graft yield for 1 hr of reaction is only 12%, whereas when the reaction is carried out for 4 hr, the graft yield increases to 18%.

**Figure 5:**
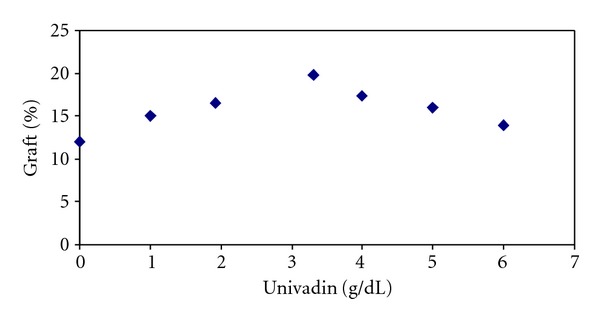
Effect of swelling agent on the grafting yield.

To improve the grafting yield, different additives and swelling agents were used. The best result was achieved with Mohr's salt (FeSO_4_·(NH_4_)_2_SO_4_·6H_2_O) with concentration of 1.92 gr/lit and with CuCl_2_ at concentration of 0.76 g/lit. This effect of swelling agent can be explained by action of Fe^2+^ ions, which partially migrates into polymer at low concentration, thereby deactivating mainly low-molecular radicals formed at the solution/polymer interphase. However, at higher concentration of Mohr's salt, Fe^2+^ ions diffusing in to the polymer phase increase. As a result, they start deactivating macroradicals to a greater extent hindering the generation and growth of graft chains. Besides, since Fe^2+^ ions accelerate the benzoyl peroxide decomposition, their high concentration favors the rapid initiator consumption. To provide a high yield of the graft PAA, a considerable concentration of macroradicals is required during reaction. Therefore, with penetration of macromolecule into the chain, the grafting will proceed with a faster pace (Table 1). When suitable swelling agent was used, grafting of acetic acid on PET fabric took place at higher speed and higher yield. Concentration of swelling agent (Univadin PB) was altered from 1 to 6 (g/lit) and its effect on the grafting yield was investigated (Figure 5). 

**Figure 6:**
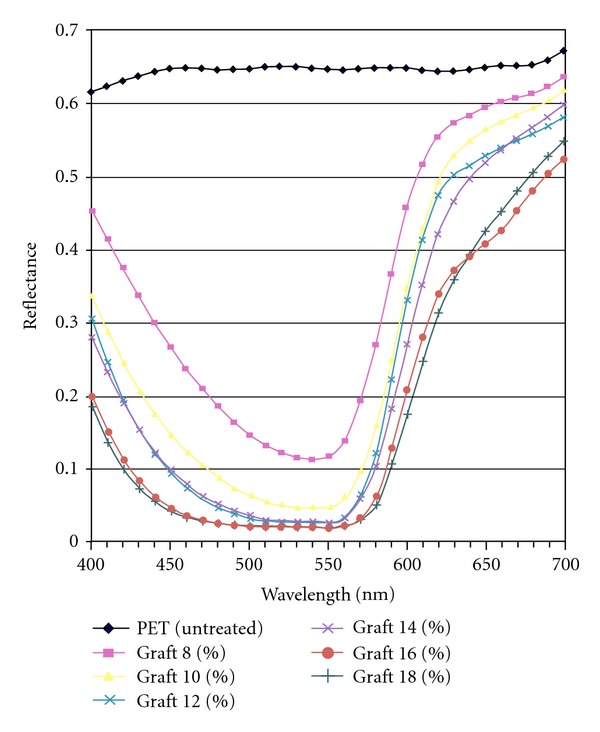
Reflectance of grafted and virgin PET.

As the concentration increases up to 4 g/lit, the graft yield increased to 17%; however, when the concentration of Univadin increased further, the yield decreased probably because after this concentration it does not have any effect on the fabric. After grafting of AA onto polyester fibers, due to the presence of carboxylic groups, the ability to absorb basic dyes increases. Polyester does not have active chemical sites therefore can not be dyed with cationic and ionic dyes. Grafted PET fabrics, however, showed good dyeing behavior with basic dyes because of carboxylic groups present in the acrylic acid. Uniform dyeing of the grafted fabric can be a good indication for uniform grafting of the carboxylic acid on the PET fabric. UV spectrophotometer data reveal that with increasing the graft percentage, the penetration and the depth of the dye into the fabric increase (Figure 6). 

**Figure 7:**
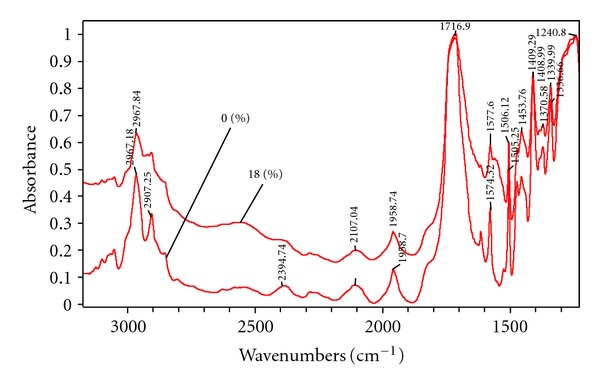
FTIR of the PET samples.

FTIR data indicate that the polyester fabric shows characteristic bands at 3432 cm^−1^ due to vibration, of free –OH groups in the end groups of PET, 2965 cm^−1^ due to methyl group, 1716 cm^−1^ due to C=O vibration, and 1015, 871, and 725 cm^−1^ due to cyclic group in PET. However, in the grafted samples, bands at 1730 cm^−1^ due to C=O and 1090 cm^−1^ due to COOH are broadened (Figure 7). This shows the increase in the concentration of carboxylic group due to the presence of acrylic acid. The grafted samples show additional bands at l540 cm^−1^ which is due to COOH group of acrylic acid. As the graft yield increases, the intensity of this band increases. Another band appears at 2930 cm^−1^ which can be assigned to C–H vibration of methane which was not present on the untreated polyester before grafting. 

**Figure 8:**
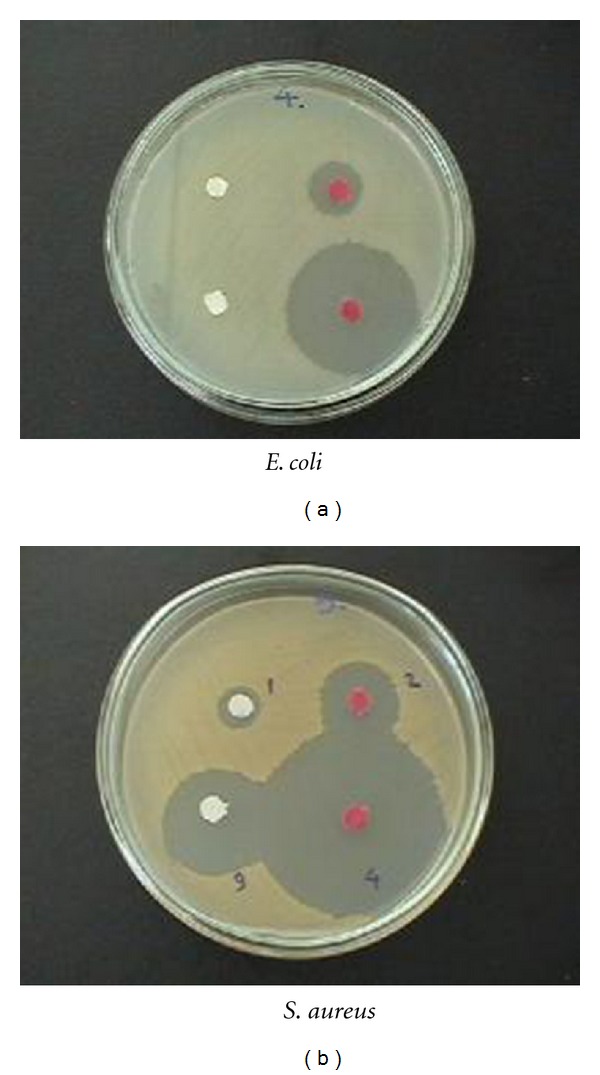
Antibacterial activity of PET fabrics determined with circle-diffusive method after 24 hr of incubation, PET washed twice (1), washed five times (3) and grafted PET (18%) washed twice (4) and five times (2).

The antibacterial assessment can be determined qualitatively from the area that the bacteria have been eradicated from, as shown in Figure 8. Antibacterial studies showed that bacterial growth of *Escherichia coli, Staphylococcus aureus, *and* Micrococcus luteus* on polyester fabric treated with different antibiotics, that is, penicillin, neomycin, and gentamycin was fast however, in the grafted samples, this growth was slowed down, and with increasing the graft percentage, the fabric shows more resistance to bacterial growth. 

Irgacide Lp-10 was used as an antimicrobial agent. The fabrics were soaked with Irgacide and washed and treated with bacteria, and its effect on the bacterial growth was investigated. The diameter of inhibition ring of untreated PET fabric after twice washing was zero which means that the remained amount of Irgacide is so less that it could not prevent the growth of *E. coli* and *M. luteus* but *S. aureus* being a gram-positive bacteria and more sensitive even with a very little amount of Irgacide could grow at all. With 5 times washing, it had no effect on the bacterial growth, and all the bacteria could show relatively good growth. The diameter of the inhibition zone of bacterial growth for grafted samples on which *E. coli* was cultured was 30.6 mm and for *S. aureus* it was 46.7 mm and for *M. luteus* 10.7 mm. Therefore, acrylic acid grafted on PET make strong bands with Irgacide which do not let the bacterial growth take place even after 5 times washing. Irgacide showed the most resistance to *S. aureus* followed by *E. coli* and *M. luteus.*

**Figure 9:**
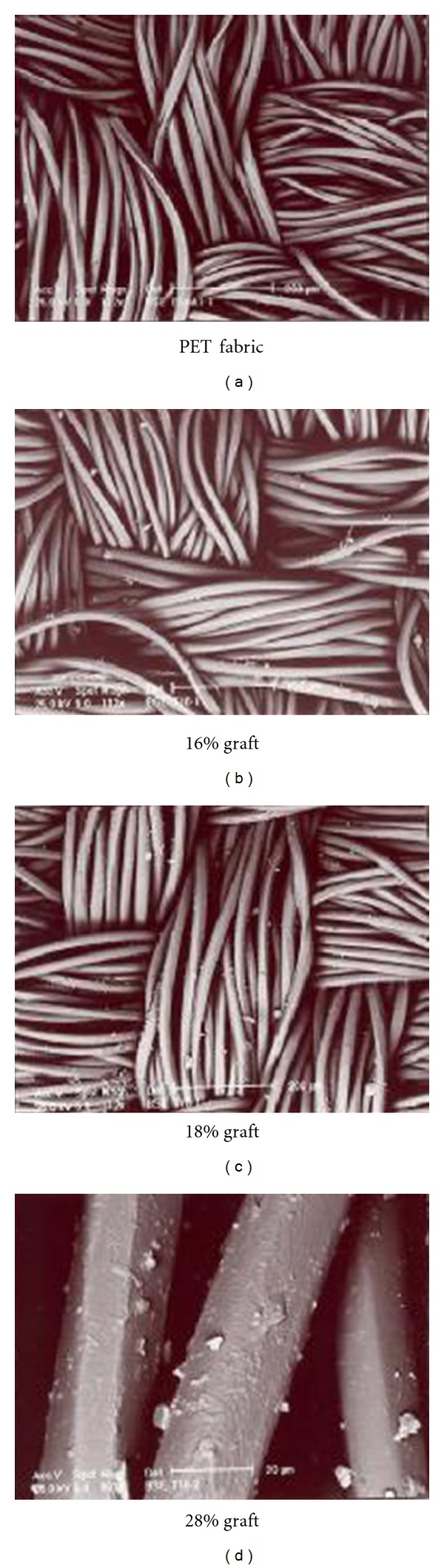
SEM photographs of the PET and grafted PET fabrics.

SEM photographs of the grafted fibers show that the surface of the polyester fibers is smooth; however, after grafting, it becomes uneven, and the growth of graft can be seen on the surface of the fibers (Figure 9). The distinction is attributed to the fragmentation of polymer chains caused by the surface etching and the deposition of the grafted components on the filament surface. This is a general behavior and has been observed in other systems as well [19]. 

## 4. Conclusions

Poly(ethylene terephthalate) was grafted with acrylic acid using benzoyl peroxide and using different procedures, and the effect of graft copolymerization on the graft yield has been studied. Three antibiotics, namely, penicillin, neomycin, and gentamycin, were attached to the grafted fabrics, and cell cultures were carried out on the fabrics. With increasing the concentration of the initiator (BP), the grafting percentage increases and reaches a max value of 18% with 4 g/mol of initiator. The concentration of monomer, however, affects the percentage of the grafting, and with increasing the monomer concentration up to the 2 lit/mol, grafting percentage increases. The effect of other parameters such as time, temperature, swelling agents, and concentration of Univadin PB has also been studied. Samples were dyed with Maxillon Red GRL (CI Basic Red 46). The grafted samples showed better dyeing behavior and could be easily dyed. The grafted samples showed good antibacterial properties.

## Figures and Tables

**Table 1:** Effect of salt additives on the acrylic acid grafting percentage.

Mohr's saltconcentration (g/dL)	Grafting percentage	Cupper chloride concentration (g/dL)	Grafting percentage
0.76	13.5	0.38	17.64
1.15	13.71	0.76	19.73
1.53	14.6	1.15	17.2
1.92	16.52	1.53	17
2.7	14.33	2.3	15
